# Influence of d-glutamine and d-glutamic acid sequences in optical peptide probes targeted against the cholecystokinin-2/gastrin-receptor on binding affinity, specificity and pharmacokinetic properties

**DOI:** 10.1186/2191-219X-3-75

**Published:** 2013-11-15

**Authors:** Susanne Kossatz, Rosalba Mansi, Martin Béhé, Peter Czerney, Ingrid Hilger

**Affiliations:** 1 Department of Experimental Radiology, Institute of Diagnostic and Interventional Radiology I, Jena University Hospital-Friedrich Schiller University Jena, Erlanger Allee 101, Jena, 07747, Germany; 2 Institute for Nuclear Medicine, University Hospital Freiburg, Hugstetter Strasse 55, Freiburg, 79106, Germany; 3 Center for Radiopharmaceutical Sciences ETH-PSI-USZ, Paul Scherrer Institute, Villigen-PSI, 5232, Switzerland; 4 Dyomics GmbH, Otto-Schott-Strasse 15, Jena, 07745, Germany

## Abstract

**Background:**

Image-based diagnosis of tumours can be advanced and improved by targeted strategies addressing malignant molecular structures. A promising molecular target is the cholecystokinin-2-receptor (CCK2R) which can be targeted by high-affinity peptides called minigastrins. Here we present how the imaging properties of minigastrins tagged with near-infrared fluorescence (NIRF) dyes can be modulated by the introduction of different spacer sequences. We identify interactions of different probe variants with regard to target affinity, specificity and pharmacokinetic properties to optimize early detection of CCK2R-expressing tumours under clinical conditions.

**Methods:**

Two minigastrin probes with the same near-infrared hemicyanine fluorescence dye (DY-754) for signalling and the same CCK2R-binding peptide A-Y-G-W-M/Nle-N-F-amide but different spacers were designed as follows: ‘QE’ with three alternating d-glutamines and d-glutamic acids and ‘bivQ’ with two minigastrins, each preceded by three d-glutamines. They were tested for affinity and specificity *in vitro* on CCK2R-expressing and CCK2R-non-expressing cells. *In vivo* imaging was conducted with subcutaneous tumour-bearing nude mice after i.v. probe injection (54 to 108 nmol/kg) and under competitive conditions with non-fluorescent minigastrin (*n* = 5/group). We also assessed probe biodistribution as well as NIRF distribution in tumour sections.

**Results:**

Both probes showed high affinity and specificity to CCK2R-expressing cells *in vitro. In vivo* tumour-to-background contrasts (tumour/background ratios (TBRs) of around 6) enabled identification of CCK2R-expressing tumours by both probes with low accumulation in CCK2R-negative tumours (TBR of around 2). Specificity of the *in vivo* accumulation, revealed by competition, was higher for QE. Besides renal retention, probe uptake into organs was very low.

**Conclusion:**

The properties of optical minigastrin probes can be specifically modified by the introduction of spacer sequences. A spacer of six hydrophilic amino acids increases affinity. A mix of d-glutamic and d-glutamine acids increased target-to-background contrast. Multimerization could not increase affinity but supposedly lowered stability. The probe QE is a promising candidate for clinical evaluation in terms of diagnosis of CCK2R-expressing tumours.

## Background

Since in neoplastic disease timely diagnosis is decisive for survival, effective mechanisms for early tumour detection are of utmost importance. One strategy to improve detection is the use of targeted contrast agents, for example nanoparticles, antibodies or peptides conjugated to a radioactive element or a fluorescent dye. Particularly in endoscopical imaging of the colon, near-infrared fluorescence (NIRF) molecular imaging can benefit from the sensitivity and the real-time nature of optical imaging without being constrained by its limited penetration depth into tissue [1,2]. One receptor expressed in colorectal, gastric and other neoplasms is the cholecystokinin-2-receptor (CCK2R) [3-7]. This G protein-coupled receptor binds the regulatory peptide hormones gastrin and cholecystokinin and modified versions of them [8,9].

Peptides are an ideal basis for the development of optical imaging probes. Due to their small size of less than 100 amino acids and their high permeability, they underlie fast bioavailability and clearance [10]. Furthermore, they can display high receptor binding affinity and specificity without the immunogenicity of antibodies [11].

Peptide analogues of gastrin, the so-called minigastrins, have been shown to be very effective radiotracers for the detection and therapy of CCK2R-expressing tumours, especially medullary thyroid cancer [12-14]. In these studies, it became obvious that the addition of a one to seven amino acid spacer to the CCK2R binding sequence can modify affinity, specificity, tumour uptake and stability of the peptides [14-17].

Highest affinity and stability together with low kidney uptake have been reported for the sequence Ala-Tyr-Gly-Trp-Met-Asp-Phe-NH_2_ in combination with three or more C-terminal d-glutamines or d-glutamic acids [18], and additional enhancement of affinity was achieved by dimerization of the peptide for nuclear medicine applications [19]. Now, replacement of the radioactive element with a near-infrared dye to translate the peptide into optical probes opens up a broad field of new applications not accessible for radiotracers. Of particular interest is intraoperative imaging and fluorescence-guided endoscopy for high-resolution early cancer detection in colorectal cancer screenings.

Our group recently showed that a minigastrin consisting of a six d-glutamine spacer showed promising *in vitro* and *in vivo* characteristics for optical imaging applications [20]. In detail, we found a low nanomolar affinity (*K*_d_ = 1.8 nM), high binding specificity and selective fluorescence accumulation in CCK2R-expressing tumours in mice.

However, for successful clinical translation, a comprehensive knowledge of the main pharmacological features of minigastrin probes is fundamental. Hereto, there is an urgent need to investigate and understand the interactions of the structure of minigastrin peptides tagged with fluorescent dyes on affinity, specificity, stability and biodistribution. In detail, the relevance of the spacer lengths and the amino acid hydrophilicity and charge on probe targeting and optical imaging properties should be elucidated. Another important point is whether multivalency can improve affinity also in optical minigastrins as it has already been shown for nuclear medicine applications.

To investigate these relationships, we designed two new optical minigastrin probes with different spacer sequences. Firstly, to assess the influence of amino acid charge and hydrophilicity, we introduced a spacer of three neutral d-glutamines and three negatively charged d-glutamic acids; this strategy reduces the negative charges compared to the recently published probe [20]. Secondly, we investigated the targeting and imaging properties of a dimerized minigastrin, where a spacer of three neutral d-glutamines preceded each minigastrin molecule to gain further insight into the influence of multimerization and spacer lengths on affinity, specificity and stability.

## Methods

### Design of the optical probes

**Figure 1:**
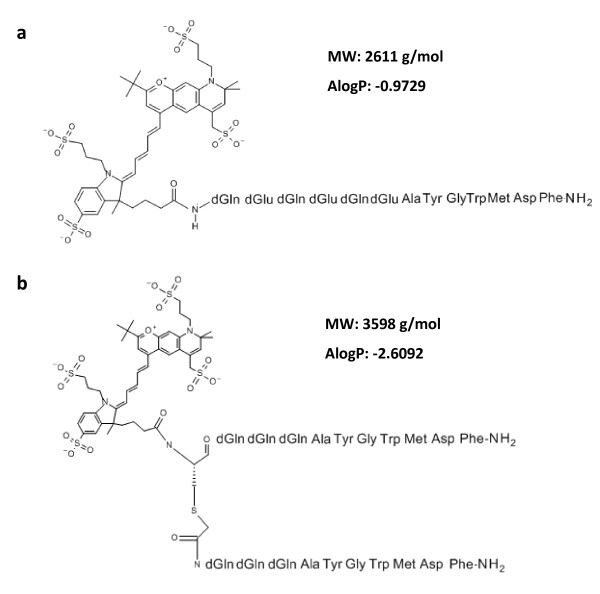
**Structural formulas of optical CCKR binding peptide probes. (a)** The probes differed in the spacer sequence connecting the NIR fluorochrome DY-754 and the CCK2R binding peptide. The probe QE displayed a spacer of three negatively charged d-glutamic acids alternating with three d-glutamines. **(b)** The probe bivQ had a bivalent nature, where each of the two minigastrin sequences was preceded by a spacer of three d-glutamines. Structural formulas were drawn with SymyxDraw.

Two variants of NIRF cholecystokinin-2-receptor targeting peptide probes were fabricated. Both were based on the CCK2R binding peptide sequence Ala-Tyr-Gly-Trp-Met/Nle-Asp-Phe-amide that shows high affinity to the CCK2R [21,22], but differed in the N-terminal amino acids (‘spacer sequence’) to investigate the influence of different spacer variants on affinity, specificity and pharmacokinetic properties of the peptides. The spacer of probe QE consisted of three alternating neutral d-glutamines and negatively charged d-glutamic acids each [13,17,23] (Figure 1a). The probe bivQ had a bivalent character, in which two minigastrin sequences, each of them consisting of a spacer of three d-glutamines, were connected to the fluorochrome (Figure 1b). We calculated ALogP values of the compounds using the fragmental method employed in the SymyxDraw software [24]. Peptides were synthesized, purified and lyophilised (Peptide Specialty Laboratories, Heidelberg, Germany), followed by conjugation to the *N*-Hydroxysuccinimid of the NIR fluorochrome DY-754 (Dyomics GmbH, Jena, Germany; absorption 748 nm, emission 772 nm). We selected the fluorochrome DY-754 due to its low non-specific binding to tissue and renal elimination properties as it has been described previously [20].

### Cell culture

A431/CCK2R and A431/WT, human epidermoid carcinoma cells, were a gift from Dr. Luigi Aloj (Department of Nuclear Medicine, Instituto Nazionale Tumouri, Fondazione ‘G. Pascale’ , Naples, Italy). The cells were stably transfected with either the CCK2 receptor (A431/CCK2R) or with an empty vector (A431/WT) [25]. Authenticity of the cells was confirmed by DSMZ (Deutsche Sammlung für Mikroorganismen und Zellkulturen) in 2012. Cells were grown in a monolayer culture at 37°C in a 5% CO_2_ humidified atmosphere and were maintained in D-MEM medium with 10% (*v*/*v*) FBS (Invitrogen) and passaged regularly at 70% to 80% confluency.

### Animals

Female athymic nude mice (Hsd:Athymic Nude-Foxn1^nu^ nu/nu; Harlan Laboratories, Eystrup, Germany) were housed under standard conditions with water and food *ad libitum*. Mice were maintained under a low-pheophorbide diet (C 1039; Altromin, Lage, Germany) to reduce tissue autofluorescence. All procedures were approved by the regional animal committee (reg. number 02-007/10) and were in accordance with international guidelines on the ethical use of animals. Animals were anesthetized with 2% isoflurane during all procedures. To implement subcutaneous xenografts, 5 × 10^5^ CCK2R transfected (A431/CCK2R) and non-transfected wild-type cells (A431/WT) were dispensed in Matrigel™ (BD Biosciences, Heidelberg, Germany) and injected into the right (A431/CCK2R cells) and left (A431/WT cells) flank of 8- to 12-week-old animals 10 to 15 days before *in vivo* imaging.

### Target binding in vitro

Binding of the optical probes QE and bivQ to CCK2R-expressing (A431/CCK2R) and CCK2R-non-expressing (A431/WT) cells was investigated after incubation with 0.5 μM of each probe in culture medium for 30 min at 4°C to observe active surface receptor binding and at 37°C to check for internalization behaviour, which is mandatory for *in vivo* signal amplification. Incubation with 0.5 μM of the fluorochrome DY-754 should determine the role of the interaction of the peptide with the CCK2R for probe uptake. Probe incubation was followed by cell membrane staining with wheat germ agglutinin-Alexa Fluor®-555 (WGA-555, Invitrogen, Carlsbad, CA, USA), fixation in 4% (*v*/*v*) paraformaldehyde on ice and mounting in PermaFluor® Mounting medium (Thermo Fisher, Waltham, MA, USA), containing 0.2 μg/mL Hoechst 33258 (Applichem, Darmstadt, Germany) to stain cell nuclei. Fluorescence microscopy was carried out with the digital microscope EVOSfl (AMG, Bothell, WA, USA) equipped with three filter cubes for visualization of Hoechst (excitation 335 to 379 nm, emission 417 to 477 nm), WGA-555 (excitation 511 to 551 nm, emission 573 to 613 nm) and DY-754 (excitation 690 to 730 nm, emission 752 to 798 nm).

### Binding affinity to CCK2R-expressing cells

To semiquantitatively assess affinity of the optical probes to the CCK2R, we employed an empirical method, which is based on the determination of the fluorescence intensity of cells after probe incubation according to [26]. The dissociation constant *K*_d_ was calculated from a saturation binding curve where we incubated 2 × 10^6^ A431/CCK2R cells with 0.1 to 50 μM of probe for 5 min at room temperature. Fluorescence intensities of cell pellets were measured using a NIRF small-animal scanner (Maestro®, CRi Inc., Woburn, MA, USA) following a procedure described previously by our group [20].

### Binding specificity

Determination of the binding specificity was carried out by preincubating cells with increasing concentrations of unlabelled peptide (HG-13 Minigastrin, Bachem, Weil am Rhein, Germany) ranging from 0.1 to 2,000 nM directly before adding a fixed probe concentration of 2 nM to all samples. Thereafter, fluorescence intensities of cell pellets were measured using a NIRF small-animal scanner (Maestro®, CRi) and analysed semiquantitatively according to [20].

### In vivo imaging of xenograft-bearing nude mice

Xenografts were inoculated in athymic nude mice (Hsd:Athymic Nude-Foxn1^nu^ nu/nu; Harlan Laboratories) 10 to 15 days before imaging by subcutaneous injection of 0.5 million A431/CCK2R (left flank) and A431/WT cells (right flank) dispersed in Matrigel™ (BD Biosciences). For *in vivo* fluorescence imaging, 108 nmol/kg QE or 54 nmol/kg bivQ probe was injected intravenously (i.v.), according to the results of a dose determination experiment, which was set up to identify the optimal relation between sensitivity and contrast (Additional file 1). Apart from the study group, one group of animals received the respective optical probe together with a 10-fold excess of unlabelled minigastrin to induce a competition for CCK2R binding and therefore revealing *in vivo* specificity. Another group received 108 nmol/kg of the unconjugated fluorophore DY-754 to determine dye-mediated non-specific tumour uptake, and one group remained untreated to control for probe-unrelated NIR fluorescence in the animals. At defined time points between 0 and 8 h p.i., NIRF fluorescence images (excitation 615 to 665 nm, emission >750 nm) and white light images of the animals were obtained. By means of spectral unmixing, tissue autofluorescence was removed from the images. Subsequently, fluorescence intensities (FI) were analysed over the time in regions of interest (ROIs) that were placed upon the tumours and non-tumour tissue. As a measure of contrast between tumour and non-tumour fluorescence, tumour/background ratios (TBRs) were calculated as a quotient of FI_tumour_/FI_non-tumour_.

### Biodistribution

We investigated the biodistribution of the optical probes and of the fluorochrome DY-754 to determine the influence of the dye on tumour and organ accumulation. Therefore, 8 h p.i., *ex vivo* NIRF fluorescence images of the organs and the two xenografts (A431/CCK2R and A431/WT) per mouse were taken in combination with the placement of ROIs on these organs to assess semiquantitative fluorescence intensities.

### Ex vivo probe distribution measurement

To determine intratumoural distribution of the peptide probes *ex vivo*, acetone-fixed 30-μm cryosections of explanted xenografts were scanned mesoscopically in the near-infrared scanner Odyssey® (LI-COR, Bad Homburg, Germany) at 700 nm (autofluorescence) and 800 nm (probe fluorescence).

### Immunohistochemistry

To verify the CCK2R expression in xenografts, 3-μm paraffin-embedded sections from explanted tumours were deparaffinised and antigens were demasked by microwave treatment (25 min, 200 W). Then, sections were blocked with goat serum and avidin/biotin block (Dako) before primary anti-CCK2R antibody incubation (15 ng/mL (PAB6796, Abnova, Heidelberg, Germany) in 3% BSA (*w*/*v*)) for 1 h, followed by secondary antibody incubation (0.36 μg/mL (Dianova, Hamburg, Germany) in 3% BSA (*w*/*v*)) for 1 h. Afterwards, alkaline phosphatase (AP)-conjugated streptavidin (Biozol, Eching, Germany) was used with chromogens from an AP detection system (K5005, Dako, Glostrup, Denmark) for detection. Cell nuclei were counterstained with Mayer's hematoxylin (Fluka/Sigma-Aldrich, Steinheim, Germany) and mounted with gelatine (SERVA Electrophoresis, Heidelberg, Germany).

### Statistics

Statistical significance between experimental groups was calculated using two-sample *t* test for independent samples. Significance levels were chosen as *p* < 0.05 and *p* < 0.01. All experiments were repeated at least three times, and all *in vivo* animal groups consisted of at least five animals.

## Results

### Probe binding to target and internalization in vitro

**Figure 2:**
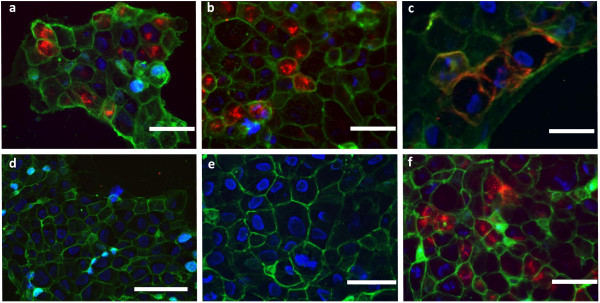
**The optical probes QE and bivQ showed specific binding and internalization in A431/CCK2R cells.** We found intracellular uptake into A431/CCK2R cells at 37°C for the probes **(a)** QE and **(b)** bivQ and **(c)** cell membrane binding at 4°C (exemplarily for bivQ). **(d)** A431/WT cells showed no probe uptake at 37°C (exemplarily for QE). **(e)** Native A431/CCK2R showed no fluorescence in the NIR spectrum. **(f)** Similar internalization into A431/CCK2R cells was observed for the probe dQ-MG-754 [20] at 37°C. Displayed are representative fluorescence microscopy images of *n* = 3 experiments. Colour coding: red, DY-754 spectrum; green, cell membrane stain WGA-555; blue, cell nuclei stained with Hoechst 33258. Bars measure 100 μm.

The probes QE and bivQ showed comparable binding and internalization at the physiologically relevant temperature of 37°C only to CCK2R-transfected A431 cells (Figure 2a,b). The involvement of active receptor binding in uptake was revealed by probe incubation at 4°C, where probe binding was confined to the cell membrane (Figure 2c). A431/WT (no CCK2R expression) cells displayed no binding of the QE and bivQ, as exemplarily shown for bivQ, confirming necessity of the CCK2R for binding (Figure 2d). Absence of NIR fluorescence in native A431/CCK2R showed that no probe-unrelated fluorescence/autofluorescence occurred (Figure 2e). Specificity of the detected fluorescence and probe-target interaction was further supported by the fact that no NIRF signal was detected in native A431/WT cells and in A431/CCK2R and A431/WT cells after incubation with the fluorochrome DY-754 (Additional file 2). Compared to the CCK2R-targeted peptide probe dQ-MG-754 [20], we detected no differences in binding and internalization properties of the probes QE and bivQ at 37°C (Figure 2f).

### Binding affinity to CCK2R-expressing cells

**Figure 3:**
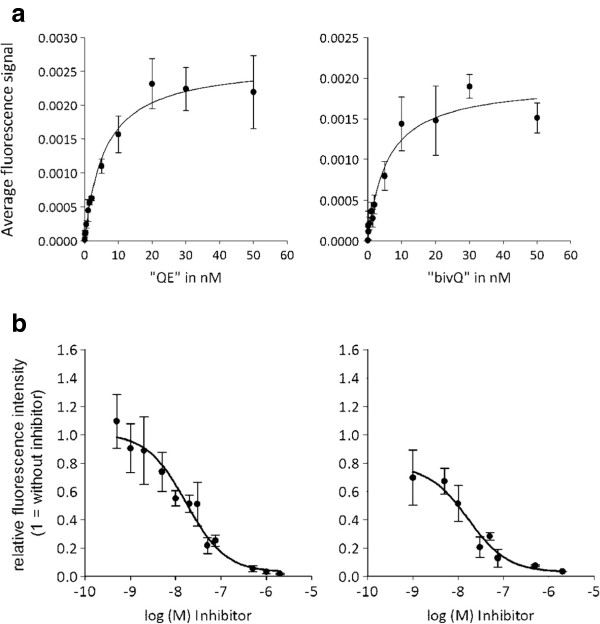
**The probes QE and bivQ showed high affinity and specificity to A431/CCK2R cells. (a)** Determination of affinity revealed dissociation constants of 5.8 ± 0.9 nM for QE (left) and 5.8 ± 1.6 nM for bivQ (right). **(b)** Specificity of CCK2R binding was evaluated after blocking with increasing amounts of non-fluorescent CCK2R ligand. A competitor concentration of 16.7 ± 0.2 nM (QE, left) and 16.6 ± 0.2 nM (bivQ, right) led to a reduction of the initial binding by 50%. Displayed are means and standard errors of three individual experiments with two parallels each.

The optical probes QE and bivQ showed a dissociation constant below the already-established high affinity threshold of 10 nM [27]. We detected identical *K*_d_ values of 5.8 ± 0.6 nM (QE) and 5.8 ± 1.6 nM (bivQ), despite the different d-glutamic acid and d-glutamine spacers and bivalency of bivQ (Figure 3a).

### Binding specificity

A high specificity of the probe-receptor interactions could be demonstrated by blocking CCK2R binding positions with increasing concentrations of non-fluorescent, competitive minigastrin before addition of a fixed concentration of one of the optical probes. Probe binding decreased with increasing concentrations of non-fluorescent minigastrin, demonstrating that the non-fluorescent minigastrin and the optical probes competed for the same, finite number of binding sites (Figure 3b). A relatively low competitor concentration of 16.7 ± 0.2 nM (QE) and 16.6 ± 0.2 nM (bivQ) led to a reduction of the initial binding by 50%, indicating that the specificity of the probes QE and bivQ was high and that no distinct influence of the different spacer compositions was detected (Figure 3b).

### In vivo imaging features of xenograft-bearing nude mice

Before starting the *in vivo* experiments, we assessed the metabolic stability of the probes against degradation by proteases (Additional file 3) and found that QE was more stable than bivQ, but both probes showed a stability against proteolytic degradation to be promising candidates for *in vivo* imaging.

**Figure 4:**
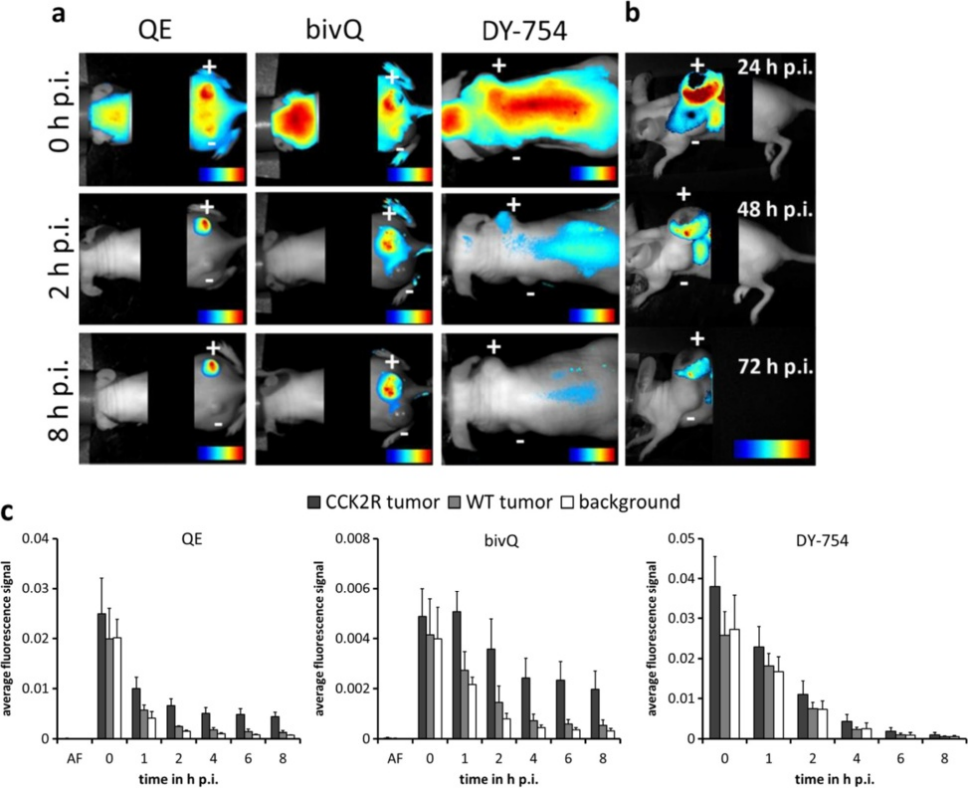
***In vivo *imaging of A431/CCK2R tumours and probe kinetics of QE and bivQ. (a)** The probes QE and bivQ clearly depict A431/CCK2R but not A431/WT subcutaneous xenografts *in vivo*. **(b)** Tumours could be detected up to 72 h after probe injection. **(c)** Semiquantitative fluorescence signal intensities from 0 to 8 h p.i. for QE, bivQ and DY-754 in A431/CCK2R tumours, A431/WT tumours and normal tissue. Means and standard deviations of *n* = 5 animals. All images were obtained with a NIRF small-animal scanner (excitation 615 to 665 nm, emission >750 nm), thresholded for their fluorescence intensity (blue, lowest FI; red, highest FI) and overlaid with their respective white light image. Plus sign, A431/CCK2R tumour; minus sign, A431/WT tumour.

Both optical probes, QE and bivQ, allowed to clearly detect subcutaneously implanted CCK2R-expressing tumours *in vivo* in nude mice, due to up to six times higher fluorescence intensity in A431/CCK2R tumours compared to non-tumour tissue (equals tumour to background ratio, TBR). A431/WT tumours, on the other hand, displayed negligible contrast between tumour and non-tumour tissue due to a lack of probe accumulation (Figure 4a). High contrasts in A431/CCK2R tumours became visible already 2 h after i.v. injection, which were comparable for both probes. The highest contrast images were captured between 2 and 8 h p.i. Although the specific tumour fluorescence signals became weaker over the time, it was easily possible to detect tumours up to 72 h after i.v. probe injection (Figure 4b). As a consequence of specific binding, fluorescence was retained in A431/CCK2R tumours, but not in A431/WT tumours and normal tissue, leading to the observed contrasts between CCK2R-expressing tumours and the background (Figure 4c). After application of the unconjugated DY-754 fluorochrome, we also observed strong fluorescence in the whole animal. However, the fluorescence from tumours and surrounding tissue decreased in the same manner, meaning that dye-mediated tumour uptake and retention of the optical probes were negligible.

**Figure 5:**
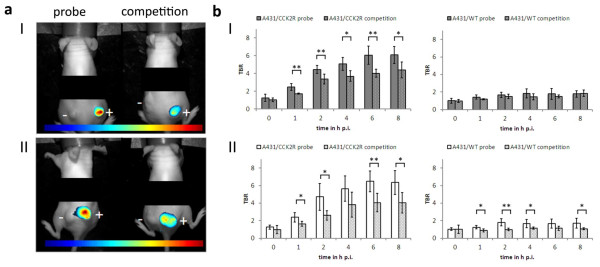
**Tumour accumulation of the probes QE and bivQ *in vivo *was highly specific. (a)** Reduction of the fluorescence signals under competitive conditions for QE (I) and bivQ (II). **(b)** TBRs significantly decreased in A431/CCK2R tumours, but not in A431/WT tumours under competitive conditions for QE (I) and bivQ (II). *p < 0.05, **p < 0.01 (Student's *t* test). Means and standard deviations of *n* = 5 animals were calculated. Images were obtained with a NIRF small-animal scanner (excitation 615 to 665 nm, emission >750 nm), thresholded for their maximum fluorescence intensity (blue, lowest FI; red, highest FI) and overlaid with their respective white light image. Plus sign, A431/CCK2R tumour; minus sign, A431/WT tumour.

Specificity of probe accumulation could be verified because *in vivo* competition of the probes with non-fluorescent minigastrin, which was injected simultaneously with the probe in a 10-fold molar excess, led to reduced fluorescence intensities in A431/CCK2R tumours (Figure 5a). The probe QE showed a high initial TBR of 6.2 ± 1.0 8 h p.i. in A431/CCK2R xenografts, and competition with non-fluorescent minigastrin reduced the TBR significantly to 4.4 ± 0.9 (*p* = 0.02). This reduction due to competition was significant (*p* < 0.05) between 1 and 8 h p.i. in A431/CCK2R tumours. A431/WT tumours displayed TBRs around the value of 2 that were not influenced by competition (Figure 5b (I)). The bivalent probe bivQ achieved TBRs comparable to QE, reaching a value of 6.3 ± 1.4 8 h p.i. that was reduced to 4.0 ± 1.2 after competition (*p* = 0.02). In this case, a significant TBR reduction could not be observed for all time points between 1 and 8 h p.i., and significance levels were lower than for QE (Figure 5b (II)). Interestingly, TBRs of A431/WT tumours also showed a significant change under competitive conditions after 1 to 4, and 8 h p.i., although the TBR values stayed at a low level between 1 and 2.

Hence, high TBRs were observed for QE and bivQ, but for QE with negatively charged d-glutamic acids, probe accumulation could be reduced more distinctly by inducing competition for specific CCK2R binding sites, showing higher specificity *in vivo* compared to bivQ.

### Biodistribution

**Figure 6:**
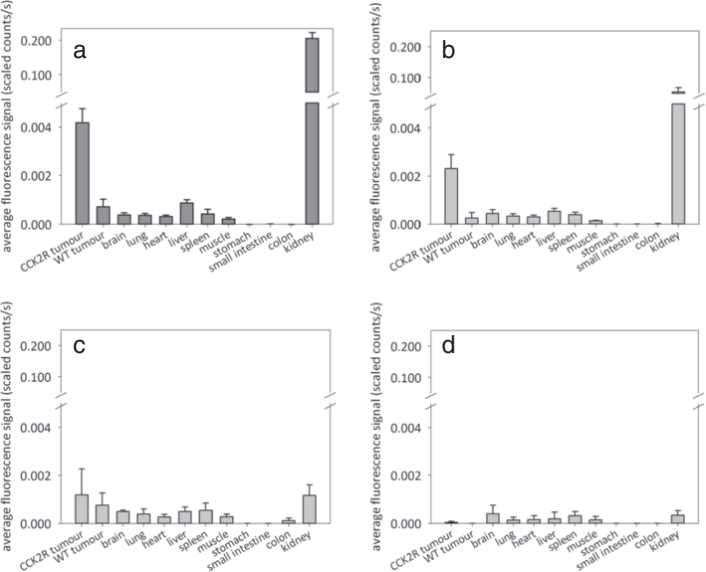
**Biodistribution of QE, bivQ and DY-754 8 h p.i. in mice carrying A431/CCK2R and A431/WT xenografts.** Semiquantitative fluorescence signals of tumours and organs injected with **(a)** QE, **(b)** bivQ and **(c)** DY-754, or **(d)** native animals. Bars represent means and standard deviations for *n* = 5 animals. *p < 0.05, **p < 0.01 (Student's *t* test of QE or bivQ versus DY-754).

Analysis of fluorescence distribution in the organs and the two xenografts in each mouse revealed strong fluorescence accumulation only in A431/CCK2R tumours and the kidneys of the animals (Figure 6a,b). In particular, the negatively charged probe QE showed a kidney accumulation that was four times higher than that of the more neutral probe bivQ, taking into account, however, that QE was injected in double amount. A weak uptake of the probe QE as well as bivQ into A431/WT tumours was also confirmed, which was comparably high for the fluorochrome DY-754 (*p* > 0.05). We observed a weakly elevated uptake of QE in the liver compared to the free DY-754. The brain, lung, heart, spleen and muscle displayed fluorescence intensities that were comparable to the respective tissue samples, which were exposed to the free DY-754 (Figure 6c) and slightly elevated compared to native animals (Figure 6d).

The stomach, small intestine and colon showed no fluorescence accumulation of the probes QE, bivQ or the fluorochrome DY-754, supporting the observation that the molecules are excreted renally and not through the hepatobiliary system (Figure 6).

### Ex vivo probe distribution in tumours

**Figure 7:**
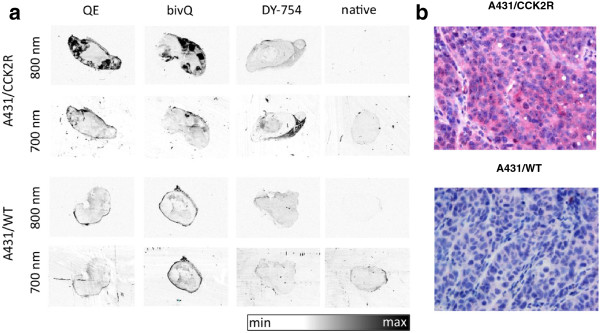
**QE and bivQ probe showing specific accumulation in vital regions of A431/CCK2R, but not of A431/WT tumours. (a)***Ex vivo* fluorescence images of tumour slices of animals that were injected with QE, bivQ and DY-754. The last column shows the native tumours. Images were obtained with an Odyssey slide scanner at 700 nm (autofluorescence) and 800 nm (probe fluorescence). All images are comparable in terms of their fluorescence intensity (white, lowest FI; black, highest FI). Representative images of three specimens/group are displayed. **(b)** Immunohistochemical staining of CCK2R in A431/CCK2R and A431/WT tumours. Red, CCK2R staining (see ‘Methods’); blue, cell nuclei staining with Mayer's haematoxylin.

The probe distribution pattern within tumours was independent from the structural properties of the probes. We detected strong fluorescence signals at 800 nm (emission maximum of the NIRF probes) for QE and bivQ only in the outer A431/CCK2R tumour regions and not in the central parts, where necrosis occurred (Figure 7a). *In vivo* application of the DY-754 fluorochrome did not result in fluorescence at 800 nm neither in A431/CCK2R nor in A431/WT tumours. Any interference with autofluorescence could be excluded since fluorescence of all tumours at 700 nm was uniform and distinctly lower than at 800 nm (Figure 7a).

The intratumoural probe distribution was also consistent with immunohistochemistry of CCK2R expression. CCK2R was expressed in the vital regions of A431/CCK2R tumours, whereas the staining was negative in A431/WT tumours (Figure 7b).

## Discussion

Our investigations revealed that the distinct spacer compositions of the probes QE and bivQ influenced all *in vivo* specificity, stability and kidney uptake above. Compared to other optical minigastrin probes, *in vivo* tumour-to-background contrast was markedly increased. The negatively charged d-glutamic acids of the probe QE led to the highest TBRs and best *in vivo* specificity and stability against proteases. The bivalent nature of the bivQ probe, however, was not adequate to increase target affinity, as observed for other multimeric small molecules.

### In vitro probe characteristics

We could confirm that binding characteristics of optical minigastrin probes are influenced by the spacer sequence, which connects the imaging moiety with the binding moiety, as it was published for radiolabelled minigastrin probes e.g. [14,28-30]. The *in vitro* experiments demonstrated that the structure of CCK2R-targeted NIRF probes QE and bivQ mainly influenced metabolic stability, whereas *in vitro* specificity was not altered. Both probes showed a high affinity binding (QE, *K*_d_ = 5.8 ± 0.6 nM; bivQ, *K*_d_ = 5.8 ± 1.6 nM). However, the minigastrin peptide with a spacer of six uncharged d-glutamines (dQ-MG-754, [20]) revealed even a higher binding affinity (*K*_d_ = 1.8 ± 0.6 nM, [20]). This is an indication that this spacer is slightly advantageous in terms of affinity compared to a spacer with a combination of three d-glutamines and three d-glutamic acids (QE) or a spacer with three d-glutamines and a bivalent peptide construct (bivQ).

Surprisingly, no increased affinity was detected for the bivalent probe bivQ, albeit multimeric variants of small molecule probes have been reported to be of superior target affinity before [31]. For example, an affinity increase was reported for RGD peptides for nuclear medicine applications, which was proportional to the number of oligomerically combined RGD units [32,33]. Also, a radiolabelled bivalent minigastrin, different from the one in this paper, showed a sixfold higher tumour uptake compared to its monovalent variant [19]. A possible influence of the dye moiety on affinity is discussed below.

Possibly, an increase in affinity by multimerization of CCK2R-targeted peptides can only be achieved by following defined basal design principles which consider that a multivalent probe has more than one possibility to bind to the target structure. It might be advantageous to choose a low-affinity minigastrin for multimerization because this increases the possibility that more than one binding event per molecule can occur before internalization prevents further multivalent interactions. Alternatively, the use of non-internalizing minigastrin antagonists might also provide a high potential to increase affinity for multivalent high-affinity probe constructs [34].

Regarding the influence of the dye conjugation on probe affinity, previously published results on analogous minigastrin variants as radioligands revealed dissociation constants in a comparably low nanomolar range. Aloj et al. 2011 [13] reported a *K*_d_ value of 9.0 ± 4.2 nM for the radiotracer analogue of QE and 4.8 ± 4.2 nM for the monovalent radiotracer version of bivQ. Hence, no loss of affinity occurred through the conjugation of the fluorochrome DY-754, opposed to former optical probes, where affinity decreased after dye conjugation compared to unconjugated or radioactively labelled peptides [11,35,36]. This is ascribed to the particular features of the fluorochrome DY-754, such as high hydrophilicity, low non-specific binding to tissue and renal elimination properties [20]. Therefore, the dye conjugation complemented probe characteristics by sterical stabilization of the molecule construct.

As we could show, the optical probes QE and bivQ bound specifically to CCK2R-expressing cells. Moreover, they were actively internalized at a temperature of 37°C but restricted to the cell membrane at 4°C. Thus, internalization behaviour was not qualitatively influenced by the different spacers and was not impeded by the dye conjugation. Successful internalization of ligand-receptor complexes has been described as a hallmark for successful tumour targeting, since retention time is prolonged and cumulative accumulation leads to signal multiplication in tumour tissue [37], while non-internalized probe molecules are cleared from blood and tissues [38,39]. These effects ultimately lead to an augmented sensitivity.

The probe QE was evaluated to be highly stable against degradation by liver proteases. After systemic application into the blood system where the protease density is lower than in liver tissue [17], the probe is likely to show up a comparatively higher stability. The probe bivQ, however, elicited only a limited metabolic stability in the presence of liver proteases. This is an indication that the metabolic stability of the probes was sufficient to reach the target site in its original constitution in our *in vivo* experiments, where we demonstrated specific targeting of CCK2R-expressing tumours. Compared to a previously published optical minigastrin probe [20] (Additional file 4), the negatively charged d-glutamic acids of the probe QE strongly improved enzymatic stability, whereas the bivalent constitution seems to increase the vulnerability of the molecule to proteases. Furthermore, the long spacer of six hydrophilic amino acids in QE seemed to be superior over the shorter three amino acid spacer in bivQ.

The high affinity binding of the probes QE and bivQ to the CCK2R in combination with internalization and hence signal amplification is an important prerequisite for low dosage for the prospective detection of CCK2R-expressing tumours in the clinical application due to a high contrast between malignant and healthy tissue. Particularly for small molecule probes, a dissociation constant of 0.1 to 10 M is needed for maximal tumour accumulation. Small molecule probes quickly achieve high tumour concentration levels but require high affinity to be retained, as unbound molecules are cleared rapidly from the tumour [40]. Due to its comparatively high metabolic stability, the probe QE elicits better perspectives for CCK2R imaging probes over the probe bivQ.

### In vivo imaging capabilities

*In vivo* small-animal imaging revealed that the new optical probes QE and bivQ were capable of high-contrast imaging of CCK2R-expressing xenografts (TBR > 6). They showed a strong specificity of tumour accumulation. The corresponding TBRs were around one-third higher TBRs than that of the previously published probe dQ-MG-754 (TBR > 4, [20]) but a comparably weak non-specific accumulation in A431/WT tumours (TBR < 2). Hence, both spacer modifications, the introduction of negatively charged d-glutamic acids in QE and the bivalency of bivQ, increased TBRs.

In terms of specificity, competitive conditions for CCK2R binding diminished the TBRs significantly in both cases, for QE and bivQ, by 30% to 40%, a high value for an only 10-fold excess of unlabelled peptide. Compared to dQ-MG-754 [20], where TBRs were reduced by 50%, competition was less complete for QE and bivQ, indicating elevated unspecific binding *in vivo*; the reasons of which remain to be identified. However, this might partly be due to the lower initial TBR of dQ-MG-754 of around 4 (compared to > 6 for QE and bivQ).

Importantly, we observed an absence of fluorescence signals in the gastrointestinal tract for QE and bivQ, resulting from the hydrophilicity-induced renal elimination pattern. The strongly negative ALogP values further support the high hydrophilicity of the molecules. A former optical minigastrin probe with a spacer of hydrophilic glutamic acids, but the less hydrophilic fluorescent dye DY-676, displayed predominantly a hepatobiliary elimination [41], underlining once more that probe characteristics are always a summation of peptide and dye properties and that selection of the dye can fundamentally change probe characteristics. With consideration of fluorescence-guided endoscopy, hepatobiliary elimination could lead to strong non-specific fluorescence signals in areas contaminated by faeces remaining after incomplete colonic irrigation, which would obscure specific fluorescence signals [36].

The renal elimination pattern that was successfully achieved by the probe design was accompanied by prominent renal retention. This was unexpected, since the radioligand variants of the probe QE as well the dye DY-754 showed a renal elimination as well but only a minimal kidney retention [20,23]. Therefore, we assume that the combination of the four negatively charged sulfo groups of the fluorochrome DY-754 together with the hydrophilicity of the peptides fostered their kidney retention. Several studies have shown that an alteration of positively or negatively charged amino acids can influence the renal uptake pattern of peptides [23,30,42]. Possibly, after glomerular filtration followed by receptor-mediated endocytosis (e.g. megalin receptor) in the proximal tubules, the probe constructs exhibit different accessibility to lysosomal degradation and hence different retention times [43]. The observation that the renal fluorescence of the probe QE was twice as high as that from the probes bivQ and dQ-MG-754 [20] implies that additional d-glutamic acids are degraded slower than d-glutamines. As long as areas other than the kidney are investigated, kidney retention will not negatively influence the imaging process. The biocompatibility of the optical minigastrin dQ-MG-754 has been already proven, where kidney cell growth *in vitro* was not diminished by the exposure to high concentrations of the probe. Accordingly, therefore, nephrotoxicity is unlikely to occur *in vivo*[20].

Our investigations have unveiled the influences of the probe constitution on imaging properties. In detail, the d-glutamines influenced especially affinity, specificity and biodistribution in a positive way. Additional d-glutamic acids can be applied to increase tumour-to-background contrast and metabolic stability, as shown here for the probe QE. Bivalency of two high-affinity minigastrins did not lead to an enhanced affinity but decreased stability. Whether these results are of general significance for peptide probes or are confined to optical minigastrins only should be elucidated in the future using other optical peptide constructs, for example for bombesin or somatostatin probes. Today, a large variety of near-infrared dyes are available, which can also be customized to possess a defined number of negative or positive charges. Hence, by selection of a defined dye and spacer, affinity and pharmacokinetic properties of small molecule probes can be selectively influenced.

## Conclusion

Our investigations revealed that the new optical probes QE and bivQ were feasible for high-contrast imaging of CCK2R-expressing xenografts in mice and showed a strong specificity of tumour accumulation. For further testing in humans, we favour the probe QE; because of the applied probe design strategies, the introduction of negatively charged d-glutamic acids favoured probe characteristics more than multimerization of the molecule. Combining two high-affinity minigastrins into a bivalent construct turned out not to further enhance affinity but to increase proteolytic degradation. Hence, multimerization of minigastrins seems to be less effective than variation of a hydrophilic six-amino acid spacer in a monovalent molecule, as shown for the probe QE. Our results help to better understand the consequences of spacer design in optical small molecule probes with the aim to build an optimal probe construct. A CCK2R-targeted optical probe with low nanomolar affinity and high specificity, renal elimination and increased metabolic stability will have the best prerequisites for reliable clinical endoscopical examinations.

## Competing interests

The authors declare that they have no competing interests.

## Authors' contributions

SK conducted the main parts of the experimental work presented in this manuscript, participated in study design, planned the presented experiments and wrote the manuscript. RM and MB were in charge of the peptide sequence conception and preliminary peptide characterization as well as in evaluation of the manuscript. PC is the inventor of the near-infrared fluorescence dye used in this study and accounted for customization of the dye construct, experiments concerning characterization of DY-754 and revision of the manuscript. IH accounts for conception of the study, planning of the experiments and revision of the manuscript. All authors read and approved the final manuscript.

## Supplementary Material

Additional file 1: **Dose determination of NIRF probes for *in vivo *experiments.** (a) Average fluorescence signals with time and (b) TBRs of CCK2R expressing tumours were determined for 4 to 216 μmol/kg i.v. injected probe. Higher probe concentrations led to higher fluorescence signals but lower specific contrasts. Lower probe concentrations led to higher contrast but were limited by sensitivity. Accordingly, the probe concentrations for *in vivo* experiments were chosen. Data represent one animal per concentration. All data were obtained with a NIRF small-animal scanner (excitation 615 to 665 nm, emission > 750 nm). Click here for file

Additional file 2: **Confirmation of specificity for CCK2R targeted probe binding by negative controls.** (a) Native A431/WT cells displayed no NIRF signal. After incubation with DY-754, neither (b) A431/CCK2R nor (c) A431/WT cells showed NIRF fluorescence. Displayed are representative fluorescence microscopy images of *n* = 3 experiments. Colour coding: red, DY-754 spectrum; green, cell membrane stain WGA-555; blue, cell nuclei stained with Hoechst 33258. Click here for file

Additional file 3: **Metabolic (protease) stability of QE and bivQ against the degradation by mouse liver homogenates.** The HPLC peak of non-degraded QE at 9.7 min elution time (a-I) decreased after 180 min of probe incubation in liver homogenate, whereas a second peak, representing degradation products, appeared at 7.2 min of elution (a-II). Degradation curves of (b) QE and (c) bivQ. The grey line in (a) displays the acetonitrile gradient (percentage in A.bidest/0.1% trifluoroacetic acid). Peaks were detected at 750 nm with a UV/VIS detector. Data represent three parallels. Click here for file

Additional file 4: **Metabolic stability of the optical CCK2R targeted minigastrin dQ-MG-754 [**[20]**].** Degradation of dQ-MG-754 for comparison with QE and bivQ. Metabolic stability against degradation by mouse liver proteases is higher than for bivQ but lower than for QE. Probe signal was detected at 750 nm with a UV/VIS detector. Data represent three parallels. Click here for file
