# Supplementary figures and images for: Influence of d-glutamine and d-glutamic acid sequences in optical peptide probes targeted against the cholecystokinin-2/gastrin-receptor on binding affinity, specificity and pharmacokinetic properties
Source: EJNMMI Res. 2013 Nov 15;3:75. doi: 10.1186/2191-219X-3-75 (PMC4176481; doi:10.1186/2191-219X-3-75)

## Slide 1
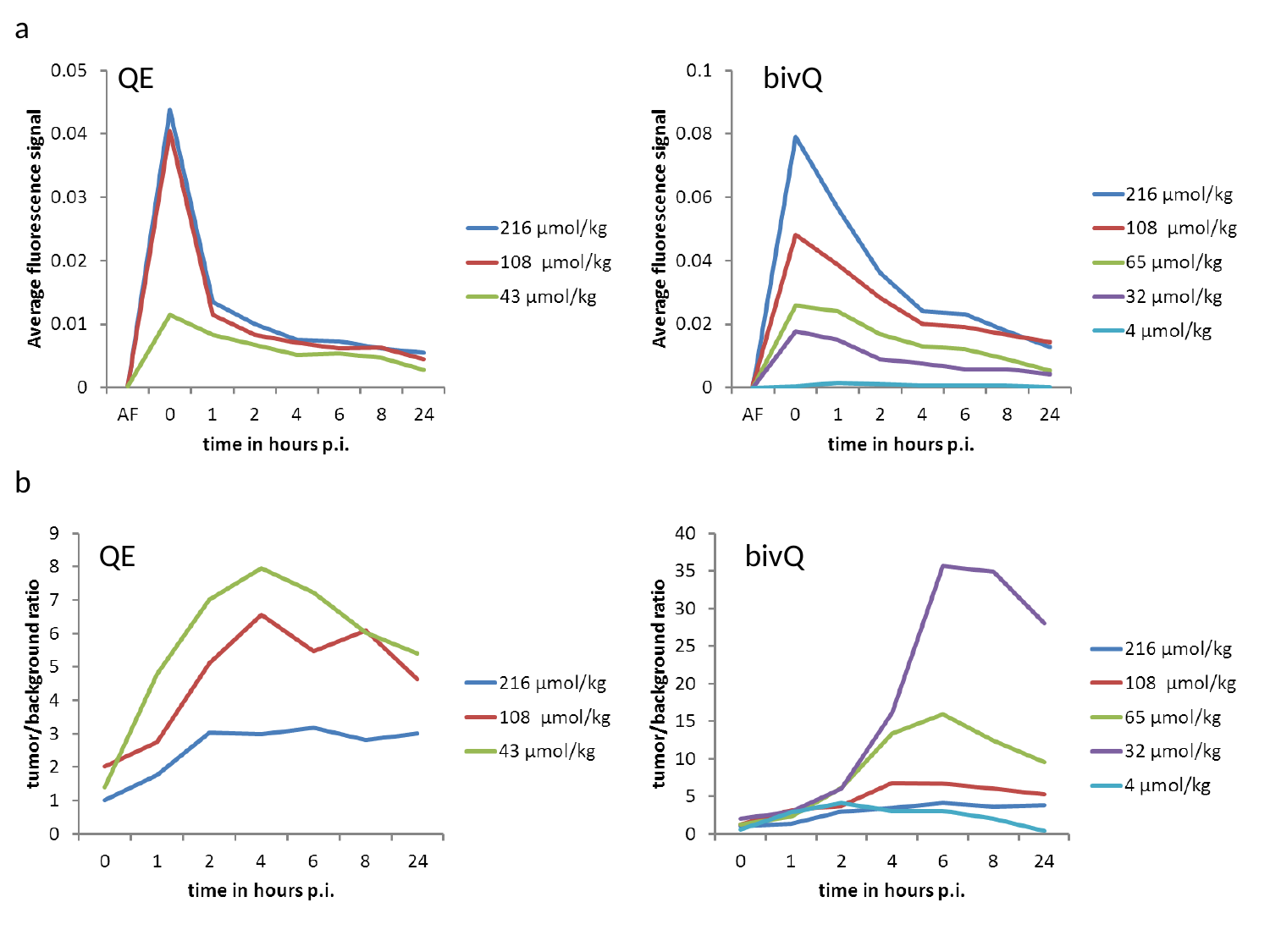

a
QE
bivQ
b
QE
bivQ

Supplement: Additional file 1: — Dose determination of NIRF probes for in vivo experiments. (a) Average fluorescence signals with time and (b) TBRs of CCK2R expressing tumours were determined for 4 to 216 μmol/kg i.v. injected probe. Higher probe concentrations led to higher fluorescence signals but lower specific contrasts. Lower probe concentrations led to higher contrast but were limited by sensitivity. Accordingly, the probe concentrations for in vivo experiments were chosen. Data represent one animal per concentration. All data were obtained with a NIRF small-animal scanner (excitation 615 to 665 nm, emission > 750 nm). [file 2191-219X-3-75-S1.pptx]

## Slide 1
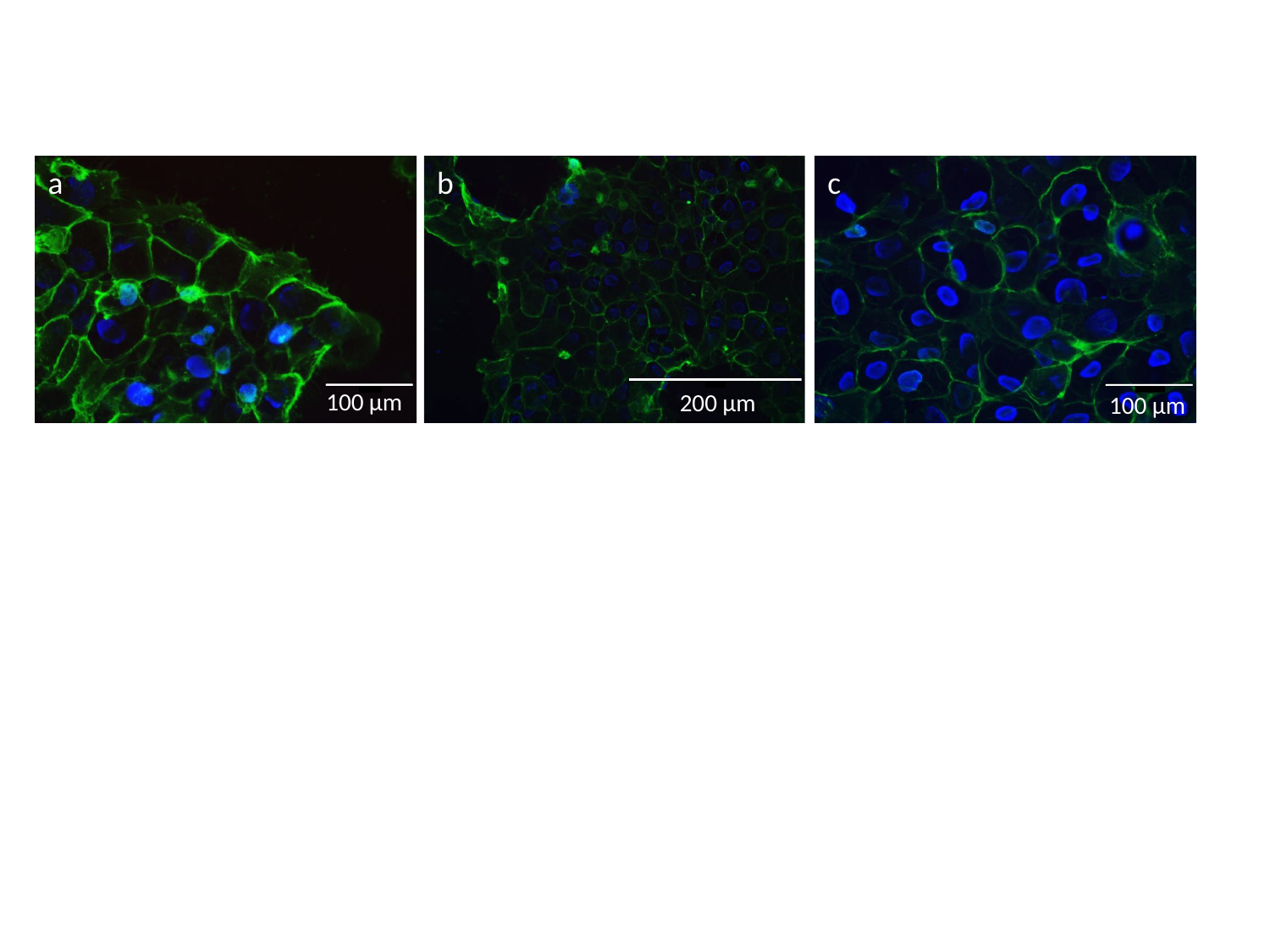

100 µm
a
200 µm
b
c
100 µm

Supplement: Additional file 2: — Confirmation of specificity for CCK2R targeted probe binding by negative controls. (a) Native A431/WT cells displayed no NIRF signal. After incubation with DY-754, neither (b) A431/CCK2R nor (c) A431/WT cells showed NIRF fluorescence. Displayed are representative fluorescence microscopy images of n = 3 experiments. Colour coding: red, DY-754 spectrum; green, cell membrane stain WGA-555; blue, cell nuclei stained with Hoechst 33258. [file 2191-219X-3-75-S2.pptx]

## Slide 1
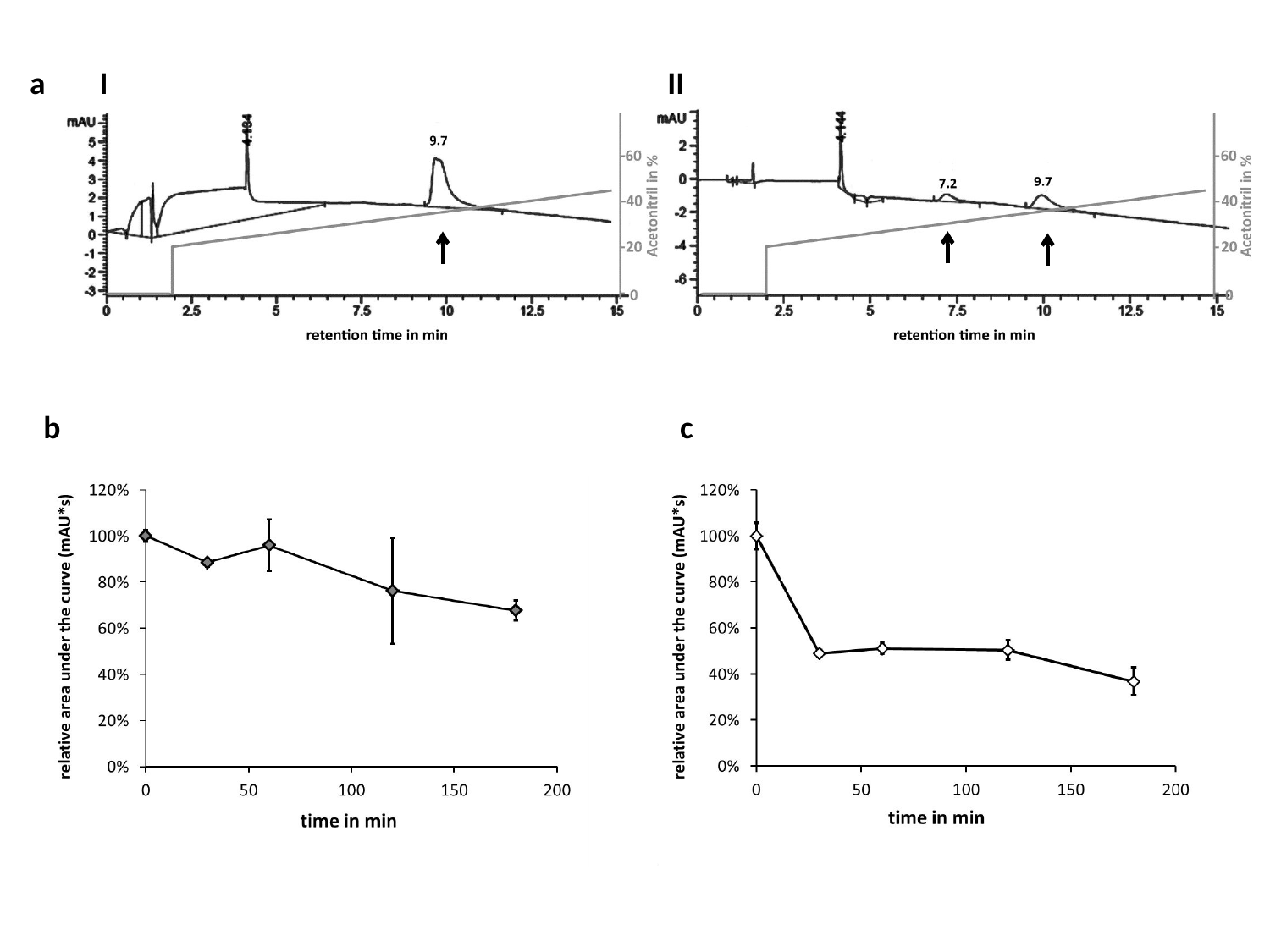

a
I
II
b
c

Supplement: Additional file 3: — Metabolic (protease) stability of QE and bivQ against the degradation by mouse liver homogenates. The HPLC peak of non-degraded QE at 9.7 min elution time (a-I) decreased after 180 min of probe incubation in liver homogenate, whereas a second peak, representing degradation products, appeared at 7.2 min of elution (a-II). Degradation curves of (b) QE and (c) bivQ. The grey line in (a) displays the acetonitrile gradient (percentage in A.bidest/0.1% trifluoroacetic acid). Peaks were detected at 750 nm with a UV/VIS detector. Data represent three parallels. [file 2191-219X-3-75-S3.pptx]

## Slide 1
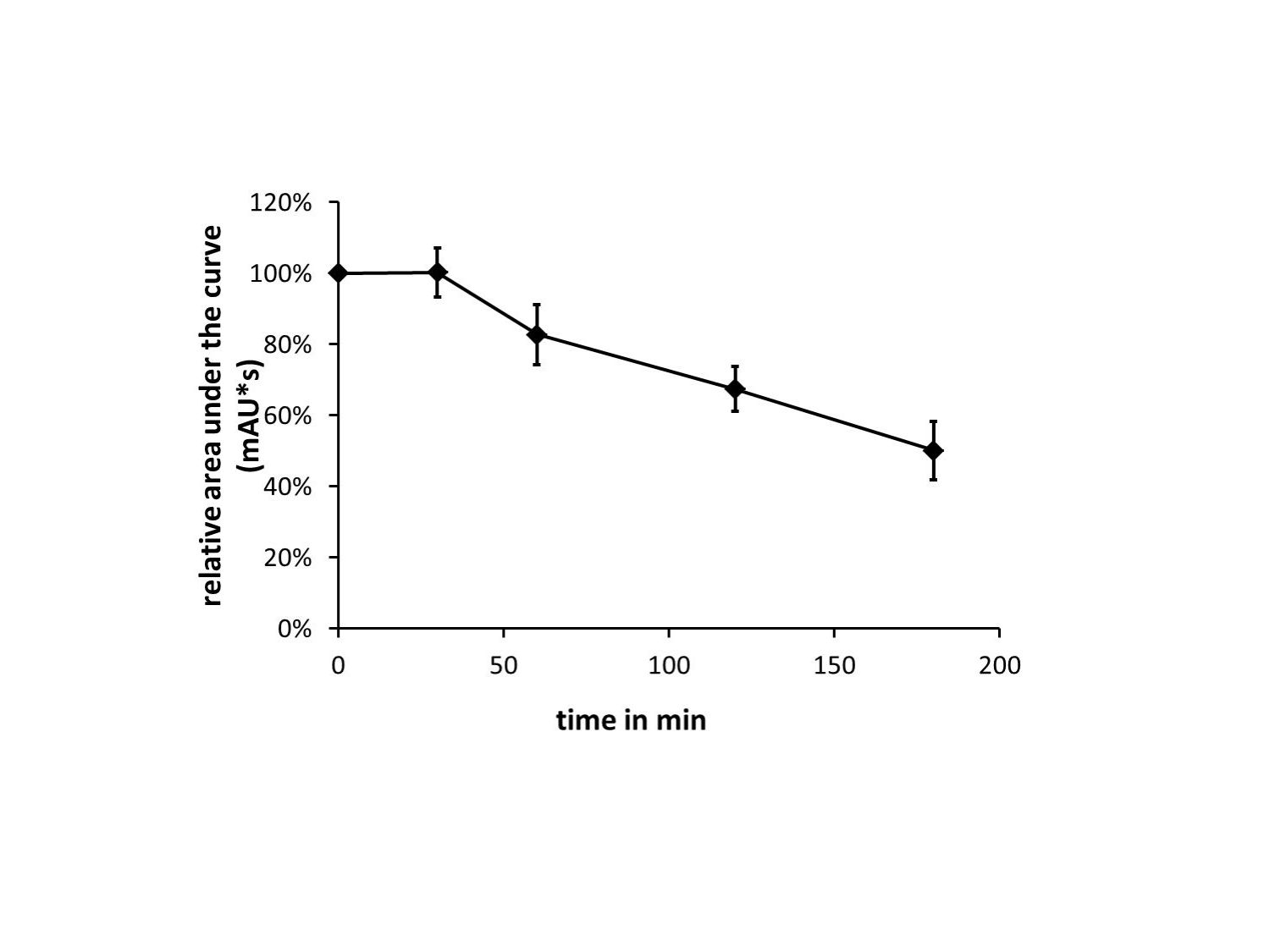

Supplement: Additional file 4: — Metabolic stability of the optical CCK2R targeted minigastrin dQ-MG-754 [[20]]. Degradation of dQ-MG-754 for comparison with QE and bivQ. Metabolic stability against degradation by mouse liver proteases is higher than for bivQ but lower than for QE. Probe signal was detected at 750 nm with a UV/VIS detector. Data represent three parallels. [file 2191-219X-3-75-S4.pptx]
